# A new paradigm for medical data circulation: research on medical data sharing based on evolutionary game theory

**DOI:** 10.3389/fpubh.2026.1854507

**Published:** 2026-06-17

**Authors:** Bingjun Guo, Dandan Wang, Muqing Niu, Jun Ma

**Affiliations:** 1 School of Business, Henan University of Science and Technology, Luoyang, China; 2 School of Computer Science and Information Engineering, Anyang Institute of Technology, Anyang, China; 3 The First Affiliated Hospital of Henan University of Science and Technology, Luoyang, China; 4 School of Art and Design, Henan University of Science and Technology, Luoyang, China

**Keywords:** evolutionary game theory, incentive mechanisms, medical examination data, sharing and mutual recognition, stability analysis

## Abstract

To address the challenge of redundant testing in medical data sharing and mutual recognition, this paper constructs an evolutionary game model involving three parties: government, medical institutions, and patients. By integrating three scenarios—pessimistic (high costs, low acceptance), baseline (cost-benefit equilibrium), and optimistic (low costs, high coordination)—the study explores the system's dynamic evolution through payoff matrix derivation, stability analysis, and MATLAB simulation. Findings indicate: Under the pessimistic scenario, the system lacks stable equilibrium, requiring external intervention to break the deadlock. In the benchmark scenario, the system converges toward weak government regulation, proactive sharing by medical institutions, and active acceptance by patients, reflecting a transition toward coordinated government regulation and market autonomy. Under the optimistic scenario, the system converges toward an ideal state of tripartite collaborative participation, fully unlocking the shared value of medical examination data. Parameter sensitivity analysis indicates that reducing sharing costs for medical institutions, regulatory costs for governments, and privacy costs for patients, while enhancing institutional network effects, are core variables driving medical examination data sharing. Based on these findings, recommendations include a tiered implementation strategy, diversified incentive mechanisms, technological empowerment, and regulatory refinement, providing theoretical foundations and practical pathways for medical examination data sharing and mutual recognition.

## 1 Introduction

In recent years, with the deepening implementation of the “Healthy China 2030” strategy, the sharing and mutual recognition of medical data—as a key component of deepening healthcare system reform—has become a vital lever for optimizing the allocation of medical resources and enhancing the efficiency of healthcare services. The Chinese government has issued a series of policy documents, including the Opinions on Deepening the Reform of the Medical and Health System (1), the Outline of the Health China 2030 Plan (2), and the Guiding Opinions on Establishing and Improving the Mutual Aid Protection Mechanism for Outpatient Services under the Basic Medical Insurance for Employees (3). These documents repeatedly emphasize, at the policy level, the importance and urgency of advancing the mutual recognition of diagnostic examination and laboratory test results. These policy documents explicitly state that advancing the interoperability and sharing of medical data will fundamentally reduce redundant examinations, alleviate patient burdens, optimize the utilization of medical resources, and build a high-quality and efficient healthcare service system. The establishment of the National Data Administration in 2023 further highlights, at the national strategic level, the strategic importance of data as a production factor that creates value through circulation (4), providing institutional safeguards and organizational foundations for the sharing and application of healthcare data.

The mutual recognition and sharing of medical examination and testing results not only concerns the efficiency of healthcare resource allocation but also directly impacts the health and wellbeing of the populace. An article titled “Analysis of the Current Status and Countermeasures for Duplicate Medical Imaging Examinations” published in the journal China Public Health indicates that the incidence rate of duplicate medical imaging examinations is 41.82% (5). This phenomenon not only exacerbates the issue of “expensive medical care” but also reflects a severe lack of coordination mechanisms within the healthcare system. From the patient's perspective, redundant examinations impose additional financial burdens and time costs, particularly for chronic disease patients and those with complex or critical conditions who require care across multiple institutions. From a societal viewpoint, redundant examinations lead to the inefficient allocation of limited medical resources, undermining the overall efficiency and quality of healthcare services.

Phenomena like “one hospital, one test” and “repeated blood draws and imaging” have long been persistent bottlenecks and pain points in public healthcare access, revealing deep-seated systemic barriers within China's medical framework. Existing research indicates that the core issue of poor medical information interoperability is a matter of conflicting interests. China's healthcare payment system predominantly operates on a fee-for-service model, where hospitals receive compensation based on the volume of services provided and tests conducted. This payment structure inherently incentivizes overtreatment and the wasteful use of medical resources. Han Pu et al. (6) employed game theory analysis to reveal that healthcare institutions face significant trade-offs in data-sharing decisions, with insufficient economic incentives and excessive liability risks being primary barriers. While mutual recognition of test results aligns with public expectations, its implementation faces formidable obstacles. This challenge is not merely technical but involves complex systemic engineering requiring the redistribution of interests among multiple stakeholders.

From an academic perspective, healthcare data sharing has garnered extensive scholarly attention. However, existing research often adopts singular viewpoints or binary frameworks, lacking systematic integration. Han Pu et al. (6) developed a two-party evolutionary game model between patients and healthcare providers from a privacy protection perspective, highlighting privacy leakage risks' impact on sharing willingness, yet omitted the critical role of government regulation. Yang Jian et al. (7) introduced blockchain technology architecture but focused on technical trust mechanisms rather than institutional coordination. Zhai Yunkai and Guo Ruifang (8) constructed a three-party evolutionary game model involving patients, healthcare institutions, and the government, yet lacked systematic comparative parameterized simulations across multiple scenarios. Jiang Jiajia (9) demonstrated that patient engagement determines the trajectory of medical data sharing. When patients widely participate in data sharing, greater value is generated, fostering a positive sharing environment. While these studies provide important foundations, they also indicate the need for more comprehensive and systematic analytical frameworks.

Technologically, advancing healthcare informatization is progressively eliminating barriers to data sharing. Emerging technologies like cloud computing, big data, and blockchain provide robust support for secure and trustworthy medical data exchange (10). Yang Jian et al. (7) explored blockchain's application in medical data sharing, noting its distributed and immutable nature facilitates establishing technical trust mechanisms. However, technological solutions alone cannot automatically resolve institutional barriers. Data sharing and mutual recognition are not merely technical challenges but complex management systems involving the redistribution of interests, risks, power, and responsibilities. The objective functions of various stakeholders—hospitals, physicians, patients, regulatory bodies, and others—are inconsistent, their decisions mutually influence each other, and they engage in typical strategic interactions. This precisely represents a classic application scenario for the game theory analytical paradigm.

## 2 Literature review

The sharing and mutual recognition of medical examination and testing data represent a critical measure for enhancing healthcare efficiency and reducing societal costs. However, its implementation faces multiple complex challenges. These challenges are not isolated but intertwined and mutually constraining, primarily falling into three systemic categories: policy regulations and trust mechanisms, technical standards and interoperability, and organizational management and economic incentives.

First, at the policy and regulatory level, although governments worldwide have introduced multiple policies to promote mutual recognition—such as China's National Health Commission's “Administrative Measures for Mutual Recognition of Medical Examination and Testing Results” (11) and the U.S. Health Insurance Portability and Accountability Act (HIPAA) (12)—significant gaps exist between policy implementation and practical outcomes. The macro framework lacks a refined quality control system and clear delineation of legal responsibilities. This creates a trust gap between medical institutions due to differences in equipment, reagents, and operational protocols. Clinicians, driven by safety concerns and liability avoidance, often opt for duplicate testing, trapping policies in a “difficult-to-implement” predicament (13). Furthermore, while the EU's General Data Protection Regulation (GDPR) sets high standards for data protection, cross-border disparities hinder the implementation of a unified mutual recognition framework, highlighting deficiencies in policy coordination and enforcement details (14).

Second, inconsistent technical standards and interoperability flaws physically obstruct data flow. Healthcare information systems remain entrenched in “data silos,” with insufficient adoption of international standards like HL7, FHIR, and LOINC. Localized proprietary formats further prevent data integration. Regional Health Information Exchange (HIE) platforms aim to integrate heterogeneous systems but face technical challenges and high costs in building unified patient indexes. Privacy-preserving technologies like homomorphic encryption and federated learning show promise but struggle to balance real-time access demands due to computational bottlenecks, further hindering sharing progress.

Finally, organizational barriers and missing incentive mechanisms fundamentally undermine willingness to share. Healthcare institutions view patient data as core assets and competitive advantages; sharing risks patient attrition, creating a lack of intrinsic motivation. The cost-benefit mismatch in data sharing—where contributors struggle to derive direct benefits—combined with unclear accountability issues (e.g., liability allocation for cross-institutional medical incidents) intensifies caution. Therefore, win-win business models and governance mechanisms must be designed, such as incentivizing sharing through preferential insurance payments or quality certification systems, to shift stakeholders' behavioral motivations.

Against this backdrop, conducting in-depth research on the multi-party game-theoretic mechanisms governing medical examination data sharing and mutual recognition holds significant theoretical and practical value. This paper constructs a tripartite evolutionary game model involving medical institutions, patients, and the government. It aims to reveal the dynamic evolutionary patterns of medical examination data sharing systems, identify key parameters influencing system equilibrium, and provide theoretical guidance and policy insights for overcoming the challenges of medical examination data sharing and mutual recognition. Specifically, this study seeks to address the following core questions: First, under what conditions will medical institutions transition from “mutual isolation” to “shared recognition”? Second, how high must the government's regulatory threshold be to effectively break the deadlock? Third, how can the “command stick” of medical insurance payments be leveraged to design more sophisticated incentive mechanisms? Fourth, as China defines data as an independent factor of production, what institutional innovations in data rights, sharing, and governance can offer globally applicable insights for digital health policy development? Through in-depth exploration of these questions, this paper aims to provide a systematic analytical framework and empirical basis for advancing medical and testing data sharing and mutual recognition. It seeks to contribute to the efficient circulation and value realization of medical data as a factor of production, thereby supporting the “Healthy China” initiative.

**Figure 1:**
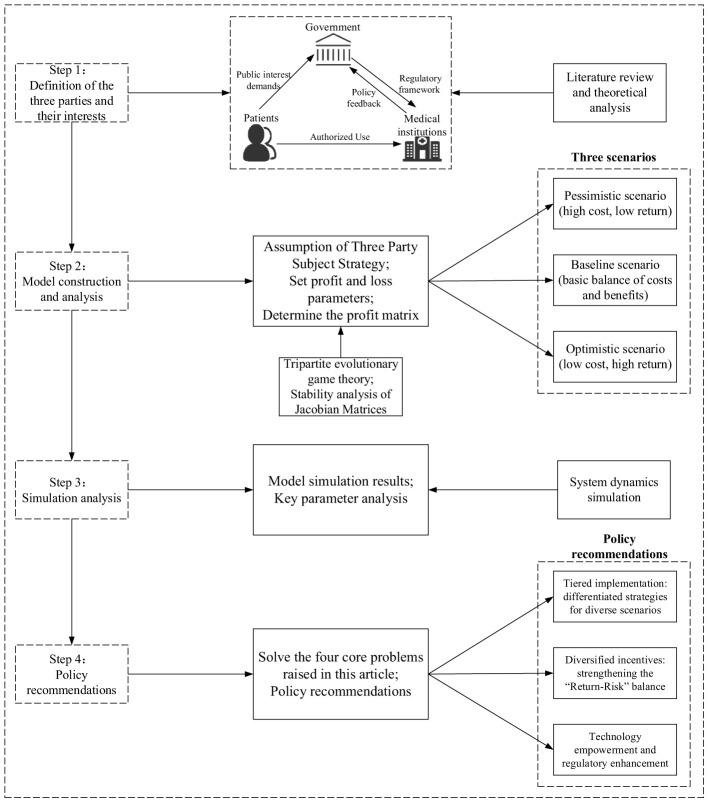
Research framework.

To achieve a balance of interests among the three parties involved in medical and testing data sharing and mutual recognition, and to explore optimal strategy choices for all stakeholders, this study constructs the research framework shown in Figure 1.

## 3 Construction of medical examination data sharing entities and game models

Medical examination data sharing and mutual recognition constitute a complex systems engineering project involving strategic interactions and behavioral coordination among multiple stakeholders. A thorough analysis of each participant's interests, behavioral logic, and interrelationships forms the foundation for constructing a scientific game model (15). This study focuses on three core entities—government, medical institutions, and patients—which collectively establish the fundamental framework for action in medical examination data sharing and mutual recognition.

### 3.1 The role of government and policy instruments

Government departments (including the National Health Commission, Medical Insurance Bureau, and Data Bureau) play multiple roles in medical examination data sharing and mutual recognition as rule-makers, incentive allocators, and dispute arbitrators. Government policy choices are influenced by multiple factors such as public interest, administrative costs, and political considerations (16). From a public interest perspective, the government focuses on optimizing the allocation of medical resources, improving overall health levels, and controlling medical expenses. From an administrative cost perspective, governments must balance regulatory investment with policy effectiveness to pursue sustainable governance models. Politically, medical examination mutual recognition, as a vital public welfare initiative, directly impacts government credibility and policy reputation through its implementation outcomes.

The government's strategic space spans {strong regulation, weak regulation}. Opting for strong regulation implies promoting sharing through institutional frameworks, economic incentives, and oversight penalties. Choosing weak regulation signifies allowing market forces to operate with minimal government intervention. Research by Guan Xin et al. (17) indicates that varying degrees of government regulation lead to distinct strategic combinations among medical institutions and patients. The level of regulation imposed on hospitals and patients determines the operational status of hospitals and the behavioral choices of patients within the market environment. Hangzhou created a favorable institutional environment by establishing a multi-departmental coordination mechanism (led by the Health Commission, with collaboration from medical insurance, finance, and data authorities) and implementing a policy mix (standard setting, platform development, performance evaluation, and incentive-constraint mechanisms), providing replicable experience for the nation (18).

### 3.2 The behavioral logic and strategic space of healthcare institutions

Healthcare institutions play a pivotal role in the sharing and mutual recognition of medical examination data, with their decision-making influenced by multiple factors including economic benefits, professional development, and management evaluations. From an economic perspective, these institutions face a profound “revenue displacement” dilemma—while data sharing can reduce redundant testing, examination and testing revenues often constitute a vital funding source for hospital operations. Professionally, data sharing enhances diagnostic efficiency and quality while aligning with medical ethics (19). However, unclear delineation of liability for medical quality and safety leads clinicians to approach mutual recognition cautiously (20). Administratively, institutions must balance the strategic value of data sharing against immediate operational pressures, with decisions often representing compromises among competing objectives.

Healthcare institutions' strategic choices primarily revolve around {sharing and mutual recognition vs. mutual isolation}. Opting for sharing and mutual recognition means actively participating in data-sharing platforms, mutually recognizing test and examination results, and avoiding duplicate tests. Choosing mutual isolation means protecting proprietary data resources, potentially increasing revenue through repeated testing. Notably, these strategic choices are not static but dynamically adjusted based on patient preferences, government policies, and anticipated benefits (21).

### 3.3 Patient motivation and behavioral patterns

As the core beneficiaries of healthcare services, patients‘ engagement and trust directly impact system effectiveness. Their behavioral decisions are influenced by three factors: economic rationality, privacy concerns, and trust levels (22). From an economic perspective, patients seek to reduce both the direct costs of redundant tests and indirect expenses such as time and transportation. Privacy concerns stem from fears that health data breaches could lead to employment discrimination, insurance denial, and social stigma, creating significant psychological barriers to participation. Gu Qiuyang et al. (23) found that privacy disclosure costs enhance user vigilance, though this positive effect is limited within certain parameters. Regarding trust levels, patients' confidence in healthcare institutions and the government directly influences their willingness to share data. Enhancing initial trust levels can significantly lower participation barriers.

Patients' strategic choices manifest as {accepting sharing, resisting sharing}. Choosing to accept sharing signifies support for data sharing and active participation within the sharing network. Opting to resist sharing indicates unwillingness to participate due to privacy concerns or other reasons, potentially leading to selection of non-sharing healthcare providers. Research by Han Pu et al. (6) reveals that patients' perceived security benefits increase their willingness to disclose private information, provided reasonable privacy protection mechanisms are in place. Notably, patients' strategic choices are influenced not only by individual factors but also closely linked to healthcare institutions' sharing practices and the intensity of government regulation, forming a complex network of behavioral interactions.

The three major actors—government, healthcare institutions, and patients—do not operate in isolation. Instead, they form systemic dynamics through complex strategic interactions, manifested as multi-level strategic dependence and behavioral feedback loops (24, 25). Healthcare institutions' willingness to share data is influenced by patient acceptance and government regulatory intensity; patient participation in decision-making depends on trust in healthcare institutions and government privacy protection measures; while the government's regulatory intensity requires adjustment based on behavioral feedback from both institutions and patients. Research by Zhai Yunkai and Guo Ruifang (8) indicates that the initial strategy choices of each actor influence the speed and outcome of strategy evolution among others. Furthermore, an optimal benefit-sharing ratio exists between patients and institutions that encourages active participation in data sharing by both parties.

This dynamic interaction creates systemic complexity in medical data sharing and mutual recognition. On one hand, the system may fall into a vicious cycle of “mutual distrust-mutual non-sharing”: medical institutions may block data due to revenue concerns, patients may resist due to privacy fears (26–30), and governments may adopt passive regulation due to high oversight costs. On the other hand, the system may enter a virtuous cycle of “mutual trust and mutual benefit-collaborative sharing”: healthcare institutions gain more patient sources through network effects (31), patients receive better experiences through convenient access to medical care, and governments enhance governance efficiency through system optimization. Understanding this system dynamics is crucial for designing effective intervention policies.

### 3.4 Model construction and parameter setting

This paper posits that the three parties—the government, healthcare institutions, and patients—are all agents with bounded rationality. Each party seeks to maximize its own interests under conditions of limited rationality, selecting corresponding strategies based on their profit-maximization objectives and the unfolding circumstances.

#### 3.4.1 Participating entities and their strategies

Within the complex system of medical examination data sharing and mutual recognition, the three core stakeholders—government, healthcare institutions, and patients—make strategic choices based on their respective interests.

1. As the representative of public interests and the provider of institutional frameworks, the government's strategy set comprises [Strong Regulation (A), Weak Regulation (B)]. Strong regulation manifests through establishing mutual recognition standards, offering economic incentives, and enforcing oversight penalties; weak regulation, conversely, reflects laissez-faire attitudes with insufficient effective intervention.
2. Healthcare institutions, as producers and users of medical data, have a strategy set of [Shared Recognition (C), Mutual Blockade (D)]. Opting for “Shared Recognition” means actively connecting to regional medical data platforms, adhering to unified data standards and quality control requirements, and acknowledging test results from other institutions. Choosing “Mutual Blockade” manifests as data siloing, potentially seeking economic gain by requiring duplicate tests.
3. Patients, as recipients of healthcare services and owners of health data, have a strategy set of [Accept Sharing (E), Resist Sharing (F)]. Accepting sharing means authorizing the use of personal health data and its flow between institutions; resisting sharing may stem from privacy concerns or distrust in data quality.

#### 3.4.2 Probability hypothesis

**Figure 2:**
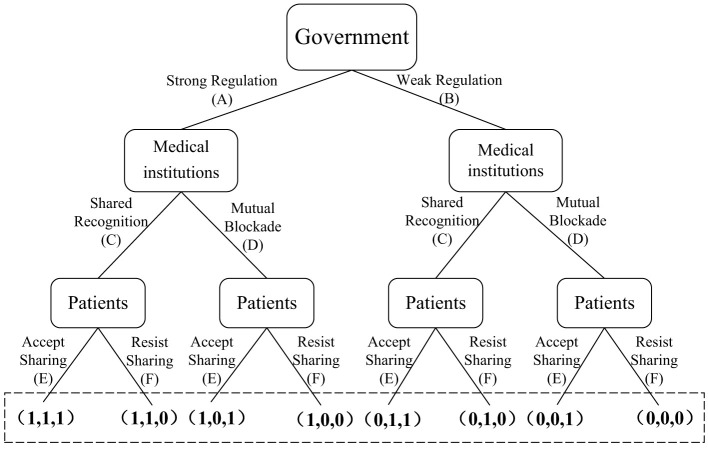
Tripartite game process.

Given the continuous choices made by the players, the strategy is uncertain. This paper assumes: The probability that the government chooses strong regulation is x (0 ≤ x ≤ 1), and the probability of choosing weak regulation is 1 − x; The probability that medical institutions choose mutual recognition and sharing is y (0 ≤ y ≤ 1), and the probability of choosing mutual lockdown is 1 − y; The probability that patients choose to accept sharing is z (0 ≤ z ≤ 1), and the probability of choosing to resist sharing is 1 − z. Based on these assumptions, this paper constructs a three-party game model involving the government, medical institutions, and patients. The game process among these parties is represented using a “game tree” method, with its terminal nodes describing equilibrium outcomes as triples (x, y, z). As shown in Figure 2.

#### 3.4.3 Profit and loss parameter setting and its revenue matrix

Based on literature review and analysis of existing mechanisms, this paper's parameter settings will consider three typical scenarios: Scenario 1(Pessimistic/High-Cost Scenario): The initial phase characterized by high initial costs for healthcare institutions to share data, significant patient privacy concerns, and challenging government oversight; Scenario 2(Baseline/Neutral Scenario): A development stage reflecting balanced costs and benefits at moderate levels; Scenario 3(Optimistic/Synergistic): A mature stage where sharing technology is established, patient acceptance is high, and sharing yields significant network effect benefits, reflecting low costs and high returns. Quantitative analysis (quantified as n, 0 ≤ *n* ≤ 100) is employed to explore the interdependent interests among stakeholders across scenarios. Benchmark values and normalization techniques are applied to intuitively analyze and highlight the core logic. Specific parameter settings are detailed in Table 1.

**Table 1:** Parameters and definitions of the tripartite evolutionary game.

Parameter symbol	Parameter meaning	Scenario 1 (pessimistic)	Scenario 2 (baseline)	Scenario 3 (optimistic)
*B*_*g*_	Direct social benefits to the government from medical data sharing and mutual recognition	30	50	70
*B*_*ge*_	Indirect social benefits to the government from medical data sharing and mutual recognition	15	25	40
*T*	Reputational benefits to the government from medical data sharing and mutual recognition	10	20	35
*C*_*g*_	Total regulatory costs to the government from medical data sharing and mutual recognition	33	23	15
*F*_*g*_	Net revenue from government fines on non-compliant medical institutions	12	20	30
*L*_*g*_	Losses from government policy failures	20	15	10
*R*_*m*_	Basic operational revenue for medical institutions without data sharing	100	100	100
*C*_*s*_	Data sharing costs for medical institutions	30	15	8
*B*_*s*_	Direct sharing benefits for medical institutions	10	20	35
*B*_*n*_	Network effect benefits gained by medical institutions through sharing	5	15	30
*L*_*d*_	Additional revenue from duplicate tests due to non-sharing	25	15	5
*S*	Sharing subsidies for medical institutions	10	20	15
*F*	Penalties for non-sharing by medical institutions	15	25	35
*R*_*p*_	Patients' baseline healthcare experience benefits	80	80	80
*B*_*p*_	Patients' direct benefits from accepting data sharing	15	25	40
*B*_*t*_	Patients' long-term health benefits after accepting data sharing	5	15	30
*C*_*p*_	Total cost to patients from privacy concerns over data sharing	40	23	11
*L*_*p*_	Patients' losses from forced non-sharing (e.g., duplicate tests)	30	30	45
*P*_*a*_	Patients' satisfaction gains from participating in data sharing	5	10	20

Based on the above parameters, a payoff matrix for the three-party evolutionary game on medical examination data sharing and mutual recognition is constructed, as shown in Table 2.

**Table 2:** Payoff matrix for the tripartite evolutionary game.

Government/medical institutions/patients			Government	
		Medical institutions	Strong regulation (*x*)	Weak regulation (1 − *x*)
Patients	Accept sharing (*z*)	Shared recognition (*y*)	*B*_*g*_ + *B*_*ge*_ + *T* − *C*_*g*_ − *S*, *R*_*m*_ + *B*_*s*_ + *B*_*n*_ + *S* − *C*_*s*_, *R*_*P*_ + *B*_*P*_ + *B*_*t*_ + *P*_*a*_ − *C*_*P*_	*B*_*g*_ + *B*_*ge*_, *R*_*m*_ + *B*_*s*_ + *B*_*n*_ − *C*_*s*_, *R*_*P*_ + *B*_*P*_ + *B*_*t*_ + *P*_*a*_ − *C*_*P*_
		Mutual blockade (1 − *y*)	−*C*_*g*_ + *F*_*g*_ − *L*_*g*_, *R*_*m*_ + *L*_*d*_ − *F*, *R*_*P*_ − *L*_*P*_	−*L*_*g*_, *R*_*m*_ + *L*_*d*_, *R*_*P*_ − *L*_*P*_
	Resist sharing (1 − *z*)	Shared recognition (*y*)	*B*_*g*_ + *T* − *C*_*g*_ − *S* − *L*_*g*_, *R*_*m*_ + *B*_*s*_ + *B*_*n*_ + *S* − *C*_*s*_, *R*_*P*_ − *L*_*P*_	*B*_*g*_ − *L*_*g*_, *R*_*m*_ + *B*_*s*_ + *B*_*n*_ − *C*_*s*_, *R*_*P*_ − *L*_*P*_
		Mutual blockade (1−*y*)	−*C*_*g*_ + *F*_*g*_ − *L*_*g*_, *R*_*m*_ + *L*_*d*_ − *F*, *R*_*P*_ − *L*_*P*_	−*L*_*g*_, *R*_*m*_ + *L*_*d*_, *R*_*P*_ − *L*_*P*_

## 4 Tripartite evolutionary game equilibrium analysis of medical examination data sharing

### 4.1 Analysis of the benefits of the three parties involved in the game

Within the evolutionary game framework, the expected payoffs of each participating entity determine the direction of their strategy adjustments. Based on the payoff matrix in Table 2, the expected payoff functions for the three parties under mixed strategies can be derived. Let the expected payoffs for the government sector choosing the “Strong Regulation” and “Weak Regulation” strategies be denoted as *U*11 and *U*1_2_, respectively. Then, the average expected payoff *U*1 is:


$$\begin{array}{l} U_{11}=yz\left(B_{g}+B_{ge}+T-C_{g}-S\right)+y\left(1-z\right)\left(B_{g}+T-C_{g}-S-L_{g}\right)+\left(1-y\right)z\left(-C_{g}+F_{g}-L_{g}\right)+\left(1-y\right)\left(1-z\right)\left(-C_{g}+F_{g}-L_{g}\right) \end{array}$$ (1)

$$\begin{array}{l} U_{12}=yz\left(B_{g}+B_{ge}\right)+y\left(1-z\right)\left(B_{g}-L_{g}\right)+\left(1-y\right)z\left(-L_{g}\right)+\left(1-y\right)\left(1-z\right)\left(-L_{g}\right) \end{array}$$ (2)

$$\begin{array}{l} U_{1}=xU_{11}+(1-x)U_{12}=x\{yz(B_{g}+B_{ge}+T-C_{g}-S)+y(1-z)(B_{g}+T-C_{g}-S-L_{g})+(1-y)z(-C_{g}+F_{g}-L_{g})\\ +(1-y)(1-z)(-C_{g}+F_{g}-L_{g})\}+(1-x)\{yz(B_{g}+B_{ge})+y(1-z)(B_{g}-L_{g})+(1-y)z(-L_{g})+(1-y)(1-z)(-L_{g})\} \end{array}$$ (3)

Similarly, let the expected payoffs for healthcare institutions choosing the “Shared Recognition” and “Mutual Blockade” strategies be denoted as *U*_21_ and *U*_22_, respectively. Then the average expected payoff *U*_2_ is:


$$\begin{array}{r} U_{21}=zx\left(R_{m}+B_{s}+B_{n}+S-C_{s}\right)+z\left(1-x\right)\left(R_{m}+B_{s}+B_{n}-C_{s}\right)+\left(1-z\right)x\left(R_{m}+B_{s}+B_{n}+S-C_{s}\right)+\left(1-z\right)\left(1-x\right)\\ \left(R_{m}+B_{s}+B_{n}-C_{s}\right) \end{array}$$ (4)

$$\begin{array}{l} {\;U}_{22}=zx\left(R_{m}+L_{d}-F\right)+z\left(1-x\right)\left(R_{m}+L_{d}\right)+\left(1-z\right)x\left(R_{m}+L_{d}-F\right)+\left(1-z\right)\left(1-x\right)\left(R_{m}+L_{d}\right) \end{array}$$ (5)

$$\begin{array}{r} U_{2}=yU_{21}+(1-y)U_{22}=y\{zx\left(R_{m}+B_{s}+B_{n}+S-C_{s}\right)+z\left(1-x\right)\left(R_{m}+B_{s}+B_{n}-C_{s}\right)+\left(1-z\right)x\left(R_{m}+B_{s}+B_{n}+S-C_{s}\right)\\ +(1-z)(1-x)(R_{m}+B_{s}+B_{n}-C_{s})\}+(1-y)\{zx(R_{m}+L_{d}-F)+z\left(1-x\right)(R_{m}+L_{d})+\left(1-z\right)x(R_{m}+L_{d}-F)\\ +(1-z)(1-x)(R_{m}+L_{d})\} \end{array}$$ (6)

Let the expected payoffs for the patient choosing the “Accept Sharing” and “Resist Sharing” strategies be denoted as *U*31 and *U*3_2_, respectively. Then the average expected payoff *U*3 is:


$$\begin{array}{l} U_{31}=yx\left(R_{P}+B_{P}+B_{t}+P_{a}-C_{P}\right)+y\left(1-x\right)\left(R_{P}+B_{P}+B_{t}+P_{a}-C_{P}\right)+\left(1-y\right)x\left(R_{P}-L_{P}\right)+\left(1-y\right)\left(1-x\right)\left(R_{P}-L_{P}\right) \end{array}$$ (7)

$$\begin{array}{l} U_{32}=yx\left(R_{P}-L_{P}\right)+y\left(1-x\right)\left(R_{P}-L_{P}\right)+\left(1-y\right)x\left(R_{P}-L_{P}\right)+\left(1-y\right)\left(1-x\right)\left(R_{P}-L_{P}\right) \end{array}$$ (8)

$$\begin{array}{l} U_{3}=zU_{31}+\left(1-z\right)U_{32}=z\{yx(R_{P}+B_{P}+B_{t}+P_{a}-C_{P})+y(1-x)(R_{P}+B_{P}+B_{t}+P_{a}-C_{P})+(1-y)x(R_{P}-L_{P})\\ +(1-y)(1-x)(R_{P}-L_{P})\}+(1-z)\{yx(R_{P}-L_{P})+y(1-x)(R_{P}-L_{P})+(1-y)x(R_{P}-L_{P})+(1-y)(1-x)(R_{P}-L_{P})\} \end{array}$$ (9)

### 4.2 Stable strategy analysis of the evolution of the three parties in the game

Based on the profit analysis of the aforementioned three parties, the replication dynamic analysis method yields the following:

(1) The government's replicating dynamic equation is expressed as:


$$\begin{array}{l} F\left(x\right)=\frac{dx}{dt}=x\left(U_{11}-U_{1}\right)=x\left(1-x\right)\left[y\left(T-C_{g}-S-L_{g}\right)-C_{g}+F_{g}\right] \end{array}$$ (10)

(2) The replicating dynamic equation for medical institutions is expressed as:


$$\begin{array}{l} F\left(y\right)\;=\;\frac{dy}{dt}=y\left(U_{21}-U_{2}\right)=\;y\left(1-y\right)\left[\left(B_{s}+B_{n}-C_{s}-L_{d}\right)+x\left(S+F\right)\right] \end{array}$$ (11)

(3) The patient's replication dynamic equation is expressed as:


$$\begin{array}{l} F\left(z\right)=\;\frac{dz}{dt}=z\left(U_{31}-U_{3}\right)=\;z\left(1-z\right)y\left(B_{P}+B_{t}+P_{a}-C_{P}+L_{P}\right) \end{array}$$ (12)

### 4.3 Model stability analysis

Furthermore, setting all three replication dynamics equations simultaneously to zero—i.e., F_(x)_ = 0, F_(y)_ = 0, F_(z)_ = 0—yields the local equilibrium points for the three-party game. Since the asymptotically stable solutions of the multi-population evolutionary game replication dynamics system must be strict Nash equilibria (32), only eight pure strategy equilibrium points need to be considered: E_1_(0,0,0), E_2_(0,0,1), E_3_(0,1,0), E_4_(0,1,1), E_5_(1,0,0), E_6_(1,0,1), E_7_(1,1,0), E_8_(1,1,1). Following Friedman's method (33), taking partial derivatives of the above replicator dynamics equations yields the Jacobian matrix *J* of the replicator system:


$$\begin{array}{l} J=\left(\begin{array}{c} \frac{\partial F\left(x\right)}{\partial x}\&\frac{\partial F\left(x\right)}{\partial y}\&\frac{\partial F\left(x\right)}{\partial z}\\ \frac{\partial F\left(y\right)}{\partial x}\&\frac{\partial F\left(y\right)}{\partial y}\&\frac{\partial F\left(y\right)}{\partial z}\\ \frac{\partial F\left(z\right)}{\partial x}\&\frac{\partial F\left(z\right)}{\partial y}\&\frac{\partial F\left(z\right)}{\partial z}\\ \; \end{array}\right)= \end{array}$$

$$\begin{array}{l} \left(\begin{array}{c} \left(1-2x\right)\left[y\left(T-C_{g}-S-L_{g}\right)-C_{g}+F_{g}\right]\&\;x\left(1-x\right)\left(T-C_{g}-S-L_{g}\right)\&0\\ y\left(1-y\right)\left(S+F\right)\&\left(1-2y\right)\left[\left(B_{s}+B_{n}-C_{s}-L_{d}\right)+x\left(S+F\right)\right]\&0\\ 0\&z\left(1-z\right)\left(B_{P}+B_{t}+P_{a}-C_{P}+L_{P}\right)\&\left(1-2z\right)y\left(B_{P}+B_{t}+P_{a}-C_{P}+L_{P}\right) \end{array}\right) \end{array}$$

Substitute E_1_~E_8_ into the Jacobian matrix, compute the eigenvalues of the matrix, and the results are shown in Table 3.

**Table 3:** Eigenvalues of the Jacobian matrix and stability conditions of equilibrium points.

Equilibrium point	Eigenvalue (λ_1_)	Eigenvalue (λ_2_)	Eigenvalue (λ_3_)	Stability conclusions
*E*_1_(0, 0, 0)	−*C*_*g*_ + *F*_*g*_	*B*_*s*_ + *B*_*n*_ − *C*_*s*_ − *L*_*d*_	0	Unstable point
*E*_2_(0, 0, 1)	−*C*_*g*_ + *F*_*g*_	*B*_*s*_ + *B*_*n*_ − *C*_*s*_ − *L*_*d*_	0	Unstable point
*E*_3_(0, 1, 0)	*T* − *C*_*g*_ − *S* − *L*_*g*_ − *C*_*g*_ + *F*_*g*_	−*B*_*s*_ − *B*_*n*_ + *C*_*s*_ + *L*_*d*_	*B*_*P*_ + *B*_*t*_ + *P*_*a*_ − *C*_*P*_ + *L*_*P*_	When all eigenvalues are less than 0, it is ESS.
*E*_4_(0, 1, 1)	*T* − *C*_*g*_ − *S* − *L*_*g*_ − *C*_*g*_ + *F*_*g*_	−*B*_*s*_ − *B*_*n*_ + *C*_*s*_ + *L*_*d*_	−*B*_*P*_ − *B*_*t*_ − *P*_*a*_ + *C*_*P*_ − *L*_*P*_	When all eigenvalues are less than 0, it is ESS.
*E*_5_(1, 0, 0)	*C*_*g*_ − *F*_*g*_	*B*_*s*_ + *B*_*n*_ − *C*_*s*_ − *L*_*d*_ + *S* + *F*	0	Unstable point
*E*_6_(1, 0, 1)	*C*_*g*_ − *F*_*g*_	*B*_*s*_ + *B*_*n*_ − *C*_*s*_ − *L*_*d*_ + *S* + *F*	0	Unstable point
*E*_7_(1, 1, 0)	−*T* + *C*_*g*_ + *S* + *L*_*g*_ + *C*_*g*_ − *F*_*g*_	−*B*_*s*_ − *B*_*n*_ + *C*_*s*_ + *L*_*d*_ − *S* − *F*	*B*_*P*_ + *B*_*t*_ + *P*_*a*_ − *C*_*P*_ + *L*_*P*_	When all eigenvalues are less than 0, it is ESS.
*E*_8_(1, 1, 1)	−*T* + *C*_*g*_ + *S* + *L*_*g*_ + *C*_*g*_ − *F*_*g*_	−*B*_*s*_ − *B*_*n*_ + *C*_*s*_ + *L*_*d*_ − *S* − *F*	−*B*_*P*_ − *B*_*t*_ − *P*_*a*_ + *C*_*P*_ − *L*_*P*_	When all eigenvalues are less than 0, it is ESS.

According to Lyapunov's First Theorem (34), an equilibrium point is asymptotically stable (ESS) only when all eigenvalues of the Jacobian matrix have negative real parts. If one or more eigenvalues have positive real parts, the equilibrium point is unstable. When the eigenvalues of the Jacobian matrix have both zero and negative real parts, the equilibrium point is in a critical state, and its stability cannot be determined based solely on the sign of the eigenvalues.

As shown in Table 3, E_3_(0,1,0), E_4_(0,1,1), E_7_(1,1,0) and E_8_(1,1,1) are the four critical equilibrium points with practical significance. At equilibrium point E_3_(0,1,0), the government adopts a weak supervision strategy, medical institutions proactively implement the mutual recognition and sharing of medical examination data results, while patients refuse to authorize the sharing of their personal health data. In this scenario, medical institutions voluntarily participate in data sharing because their net benefits from sharing are positive, whereas patients choose to resist because their privacy costs significantly outweigh the economic and health benefits derived from data sharing.

Equilibrium point E_4_(0,1,1) represents a state where the government implements weak supervision, medical institutions actively participate in data sharing and mutual recognition, and patients also voluntarily accept and authorize data sharing. This is an ideal market-driven state: the net benefits of data sharing for medical institutions exceed the revenue from repeated examinations under data blockade, and patients' net benefits from sharing also surpass their privacy costs, achieving a win-win situation for both parties. Meanwhile, the government withdraws from strong supervision as spontaneous market coordination has already achieved system stability.

E_7_(1,1,0) indicates that the government implements strong supervision; medical institutions participate in data sharing under policy pressure, but patients still choose to resist due to privacy concerns. In this case, the government enforces data sharing through subsidy and penalty measures. Medical institutions passively comply to avoid penalties or obtain subsidies, yet patients' privacy concerns have not been effectively alleviated, leading to their continued resistance.

Finally, E_8_(1,1,1) represents the ideal state of tripartite collaboration: the government maintains moderate strong supervision, medical institutions actively participate in the mutual recognition and sharing of medical examination data, and patients actively accept and engage in data sharing. This state yields extremely high social total benefits from data sharing, with relatively controllable government supervision costs and medical institutions‘ net benefits from sharing far exceeding their revenue from data blockade. Simultaneously, patients' benefits from sharing significantly exceed their privacy costs, thereby achieving a win-win outcome for all three stakeholders.

By analyzing three typical scenarios, the following conclusions are drawn:

(1) System Stability Analysis Under Pessimistic Scenarios: Substituting the parameter values from Table 1 into E_3_(0,1,0), E_4_(0,1,1), E_7_(1,1,0), and E_8_ (1,1,1).

$E_{3}\left(0,1,0\right)\{\begin{array}{c} {\;\lambda }_{1}<0\\ \lambda_{2}>0\\ \lambda_{3}>0 \end{array}\;$ ⇒ The system is unstable when the stability conditions are not satisfied.

$E_{4}\left(0,1,1\right)\{\begin{array}{c} {\;\lambda }_{1}<0\\ \lambda_{2}>0\\ \lambda_{3}<0 \end{array}\;$ ⇒ The system is unstable when the stability conditions are not satisfied.

$E_{7}\left(1,1,0\right)\{\begin{array}{c} {\;\lambda }_{1}>0\\ \lambda_{2}>0\\ \lambda_{3}>0 \end{array}\;$ ⇒ The system is unstable when the stability conditions are not satisfied.

$E_{8}\left(1,1,1\right)\{\begin{array}{c} {\;\lambda }_{1}>0\\ \lambda_{2}>0\\ \lambda_{3}<0 \end{array}\;$ ⇒ The system is unstable when the stability conditions are not satisfied.

In summary, under the pessimistic scenario, the system does not possess a stable pure-strategy equilibrium point, meaning that under the current circumstances, it is impossible to advance the process of sharing and mutual recognition of medical examination data. The reasons are analyzed as follows:

① When government departments regulate net income: *T* − *C*_*g*_ − *S* − *L*_*g*_ < 0, it indicates that regulatory costs are excessively high and government departments lack the will to enforce regulations.

② When the net revenue of medical institutions: *B*_*s*_ + *B*_*n*_ − *C*_*s*_ − *L*_*d*_ < 0, it indicates that the costs of sharing far outweigh the benefits, revealing a lack of motivation for sharing.

③ Patients exhibit low participation rates due to excessively high privacy costs *C*_*P*_.

This scenario depicts a typical challenge encountered during the early stages of promoting medical examination data sharing and mutual recognition, aligning with practices observed in certain regions of China. At this juncture, the system remains highly unstable, necessitating external intervention to break the deadlock.

(2) Analysis of system stability under the baseline scenario: Similarly, substituting the parameter values from Table 1 sequentially yields:

$E_{3}\left(0,1,0\right)\{\begin{array}{c} {\;\lambda }_{1}<0\\ \lambda_{2}<0\\ \lambda_{3}>0 \end{array}\;$ ⇒ The stability conditions are not satisfied, the system is unstable.

$E_{4}\left(0,1,1\right)\{\begin{array}{c} {\;\lambda }_{1}<0\\ \lambda_{2}<0\\ \lambda_{3}<0 \end{array}\;$ ⇒ The system is stable when the stability conditions are satisfied.

$E_{7}\left(1,1,0\right)\{\begin{array}{c} {\;\lambda }_{1}>0\\ \lambda_{2}<0\\ \lambda_{3}>0 \end{array}\;$ ⇒ The system is unstable when the stability conditions are not satisfied.

$\;E_{8}\left(1,1,1\right)\{\begin{array}{c} {\;\lambda }_{1}>0\\ \lambda_{2}<0\\ \lambda_{3}<0 \end{array}\;$ ⇒ The system is unstable when the stability conditions are not satisfied.

In the baseline scenario, where medical examination data sharing and mutual recognition are at an intermediate level of costs and benefits, reflecting a development stage of cost-benefit equilibrium, the system achieves strategic equilibrium at point E_4_(0,1,1). In the current scenario, medical institutions tend toward sharing due to positive net benefits (*B*_*s*_ + *B*_*n*_ − *C*_*s*_ − *L*_*d*_), while patients lean toward acceptance owing to positive net benefits (*B*_*P*_ + *B*_*t*_ + *P*_*a*_ − *C*_*P*_). The government, observing self-regulated sharing by institutions, gravitates toward reduced oversight.

This evolutionary path reflects a successful transition model of government guidance followed by market dominance, aligning closely with the practical approach of “policy guidance-institutional safeguards-platform support-incentive compatibility” observed in the Hangzhou case (18). Consequently, when healthcare institutions possess sufficient incentives to voluntarily share data and patients derive net benefits from sharing that motivate their participation, government authorities can adopt a relaxed regulatory strategy. This enables the system to spontaneously achieve a cooperative state without requiring further coercive intervention.

(3) Analysis of system stability under the optimistic scenario: Similarly, substituting the parameter values from Table 1 sequentially yields:

$E_{3}\left(0,1,0\right)\{\begin{array}{c} {\;\lambda }_{1}>0\\ \lambda_{2}<0\\ \lambda_{3}>0 \end{array}\;$ ⇒ The system is unstable when the stability conditions are not satisfied.

$\;E_{4}\left(0,1,1\right)\{\begin{array}{c} {\;\lambda }_{1}>0\\ \lambda_{2}<0\\ \lambda_{3}<0 \end{array}\;$ ⇒ The system is unstable when the stability conditions are not satisfied.

$\;E_{7}\left(1,1,0\right)\{\begin{array}{c} {\;\lambda }_{1}<0\\ \lambda_{2}<0\\ \lambda_{3}>0 \end{array}\;$ ⇒ The system is unstable when the stability conditions are not satisfied.

$E_{8}\left(1,1,1\right)\{\begin{array}{c} {\;\lambda }_{1}<0\\ \lambda_{2}<0\\ \lambda_{3}<0 \end{array}\;$ ⇒ The system is stable when the stability conditions are satisfied.

In the optimistic scenario, where medical testing data sharing and mutual recognition reach a mature stage characterized by advanced sharing technology and high patient acceptance, reflecting low costs and high returns, the system achieves policy equilibrium at E_8_ (1,1,1). This scenario exhibits the highest level of tripartite coordination and strongest system stability, revealing an implementation pathway of technological maturity-institutional refinement-cultural formation. It represents an ideal state where the value of data elements is fully unleashed, providing a long-term objective for medical and testing data sharing and mutual recognition. Notably, government agencies maintain oversight rather than withdrawing in this scenario because the substantial social benefits (*B*_*g*_ + *B*_*ge*_) render regulatory costs negligible.

Thus, under the ideal state of medical and testing data sharing and mutual recognition, government departments actively regulate to maintain system stability. Medical institutions, incentivized by the government, actively share data. Patients, benefiting significantly, actively participate in sharing. This creates a virtuous cycle of cooperation led by the government.

## 5 Simulation experiments and analysis

### 5.1 Initial strategy selection

To validate the theoretical model and investigate parameter effects, numerical simulations were conducted across multiple scenarios using MATLAB. By setting multiple sets of initial strategy values, the impact of different initial strategy choices by the three agents on system stability was analyzed. Initial strategy ratios were set as follows: x = y = z = 0.1, x = y = z = 0.3, x = y = z = 0.5, and x = y = z = 0.8. Simulations were conducted over the time interval [0, 1] for pessimistic, baseline, and optimistic scenarios, observing system evolution trajectories and steady-state conditions.

(1) Pessimistic scenario

**Figure 3:**
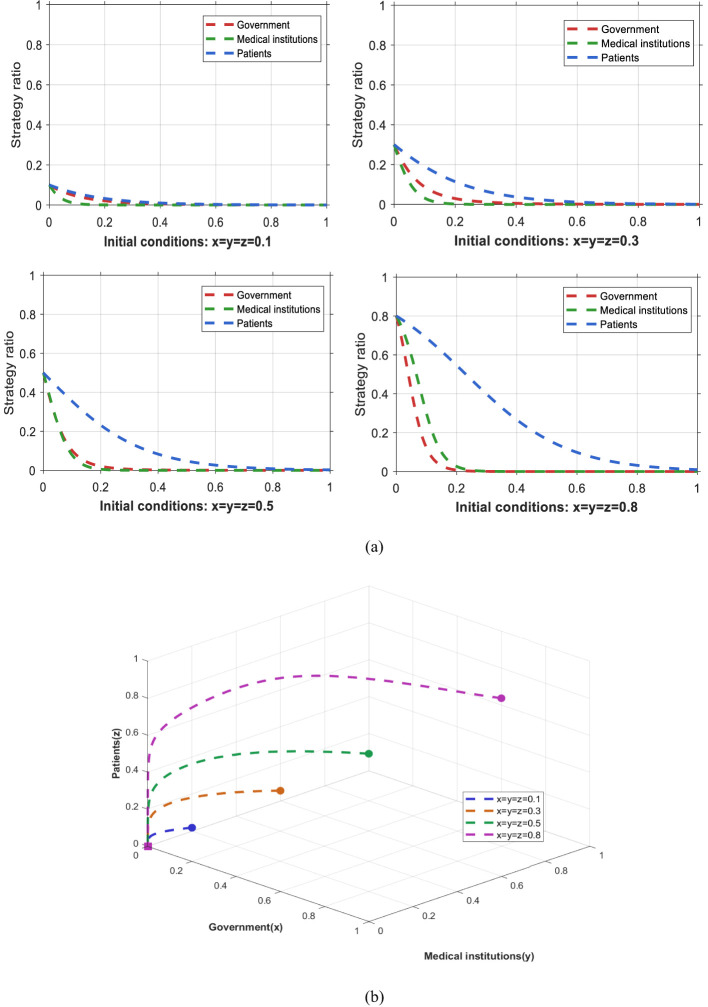
Evolutionary trend chart of initial strategy choices by three parties under pessimistic scenario.

In this scenario, healthcare institutions face high initial costs for data sharing, patients harbor significant privacy concerns, and government oversight presents considerable challenges. Consequently, the initiative to promote mutual recognition of medical examination data is encountering initial difficulties during its early implementation phase. As a result, all stakeholders exhibit low participation enthusiasm, and no consensus can be reached among the three parties. The system's evolutionary trajectory indicates that the strategy proportions of the three parties continue to oscillate without converging, lacking a stable convergence point. The simulation results are illustrated in Figure 3.

As shown in Figure 3, under the pessimistic scenario, regardless of the initial strategy selection, the participation rates of all three parties gradually approach zero over time. Furthermore, the trajectory diagram of the three-party game strategy evolution in the figure strongly corroborates this finding. Consequently, in this scenario, all parties exhibit low participation enthusiasm, leading to a stagnation in medical examination data sharing and mutual recognition. External intervention is required to break the deadlock.

(2) Baseline scenario

**Figure 4:**
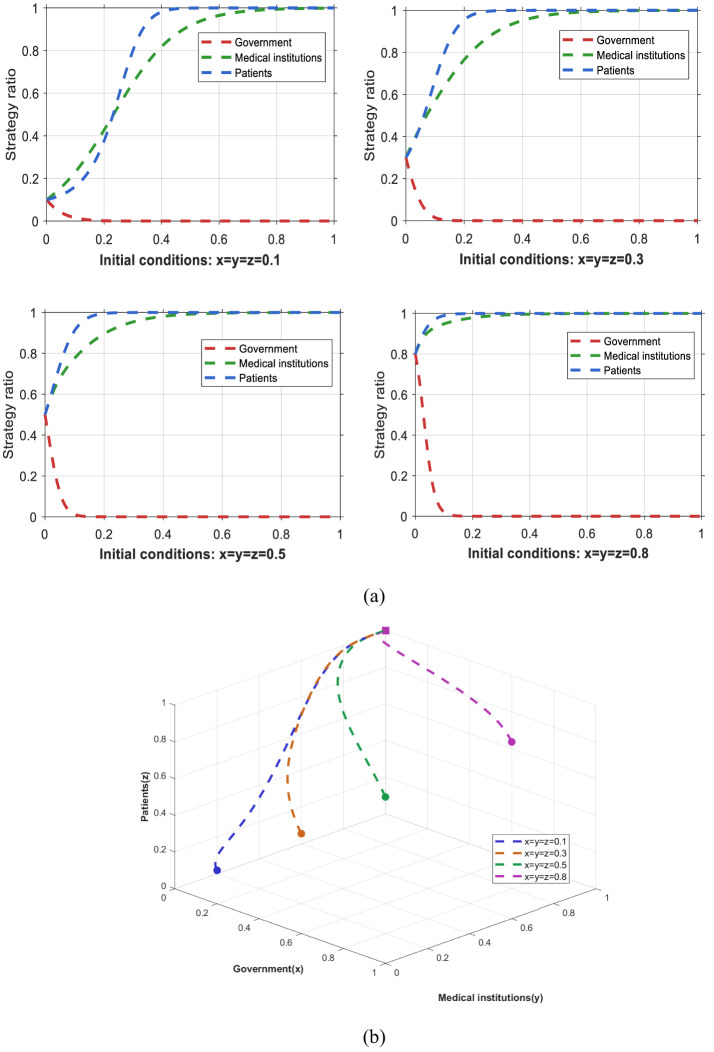
Evolutionary trend chart of initial strategy choices by three parties under baseline scenario.

In this scenario, medical examination data sharing and mutual recognition operates at an intermediate level of cost and benefit, reflecting a development stage where costs and benefits are balanced. At this point, the system achieves a strategic equilibrium at E_4_(0,1,1), meaning government departments adopt a weak regulatory approach, medical institutions actively participate in sharing and mutual recognition, and patients are willing to accept sharing. This approach enables the orderly advancement of medical examination data sharing and mutual recognition. The simulation results are shown in Figure 4.

As shown in Figure 4, under the baseline scenario, regardless of the initial strategy selection, the participation rates of medical institutions and patients gradually approach 1 over time, while the government's participation rate approaches 0. This aligns with the preceding analysis. When medical institutions have sufficient incentives to proactively share data and patients derive net benefits from sharing, thereby willing to participate, the government may adopt a relaxed regulatory approach. This enables the system to spontaneously reach a cooperative state without further intervention. The trajectory diagram of the three-party strategy evolution in the figure also converges to a stable point. Therefore, in this scenario, medical examination data sharing and mutual recognition are in a stable development phase.

(3) Optimistic scenario

**Figure 5:**
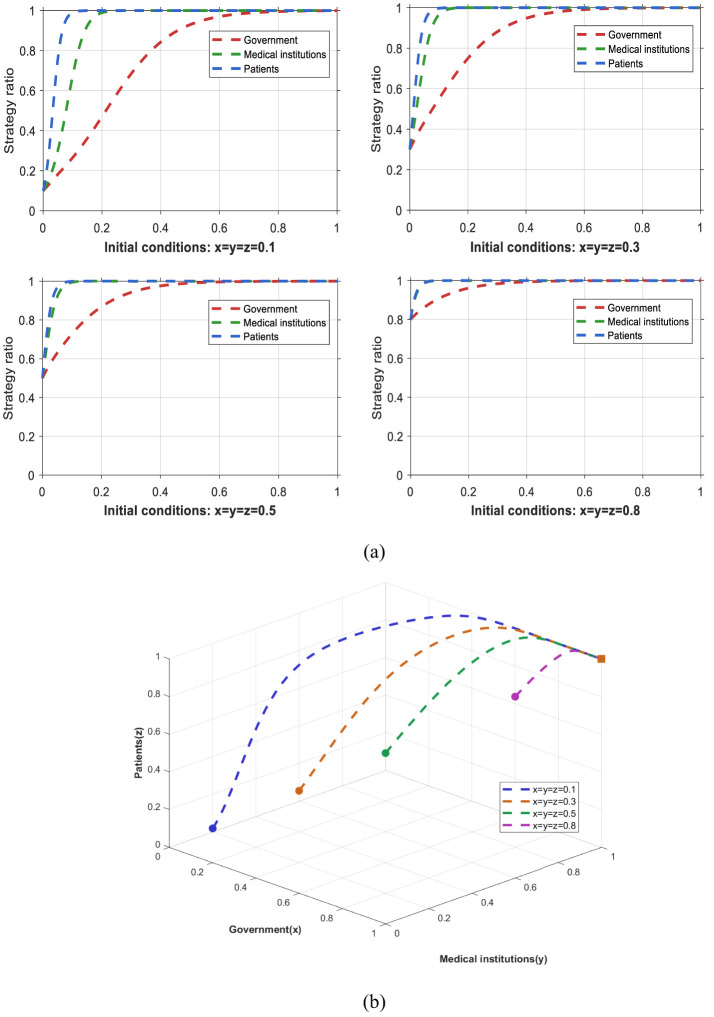
Evolutionary trend chart of initial strategy choices by three parties under optimistic scenario.

In this scenario, when medical examination data sharing and mutual recognition reach a stage of mature sharing technology and high patient acceptance, it reflects a low-cost, high-return mature phase. At this point, the system achieves policy equilibrium at E_8_ (1,1,1). This means government departments adopt strong regulatory measures, medical institutions actively participate in sharing and mutual recognition, and patients willingly accept data sharing. This scenario represents the highest level of tripartite coordination and strongest system stability, constituting the most ideal state pursued for medical examination data sharing and mutual recognition. The simulation results are shown in Figure 5.

**Figure 6:**
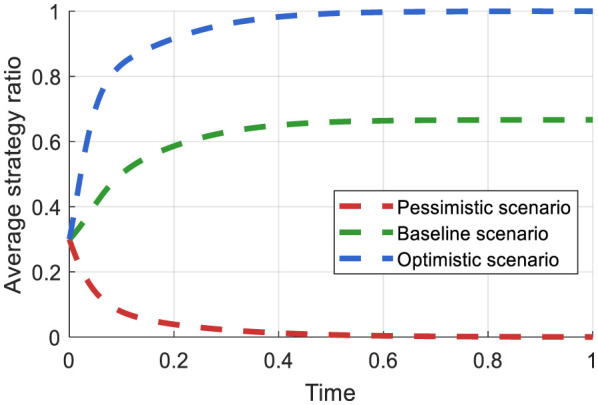
Evolution of the average strategy selection probability for the three parties under three scenarios.

As shown in Figure 5, under the optimistic scenario, the participation rates of the government, medical institutions, and patients gradually approach 1 over time, indicating that medical examination data sharing and mutual recognition have reached an ideal state. In this scenario, all parties actively engage in advancing the process of medical examination data sharing and mutual recognition, maximizing the value of data elements. Additionally, the evolutionary trends of average strategy selection probabilities across the three scenarios are illustrated in Figure 6.

Figure 6 visually illustrates the dynamic characteristics of the medical testing data sharing system throughout its evolution from an initial state of difficulty to an ideal state: In the pessimistic scenario, the proportion of strategies oscillates persistently at low levels, reflecting the high instability during the system's initial launch phase. In the baseline scenario, the strategy steadily converges to a moderate level, reflecting a gradual development path driven by the synergistic effects of government guidance and market mechanisms; In the optimistic scenario, the strategy rapidly converges to a high level, showcasing the ideal state of synergy achieved through technological maturity and institutional refinement. This not only validates the rationality of the evolutionary game model but also provides crucial evidence for policymakers to identify critical intervention points and evaluate policy effectiveness.

### 5.2 Key parameter analysis

In this study, key parameters influencing system evolution include healthcare institution data sharing costs *C*_*s*_, government regulatory costs *C*_*g*_, direct sharing benefits *B*_*s*_ for institutions, patient privacy costs *C*_*P*_, subsidy income *S* for sharing institutions, and penalty *F* for non-sharing institutions. With the initial strategy ratio set at x = y = z = 0.3, the analysis of how these parameters affect the stability of the evolutionary system under different scenarios is presented below.

(1) Pessimistic scenario

**Figure 7:**
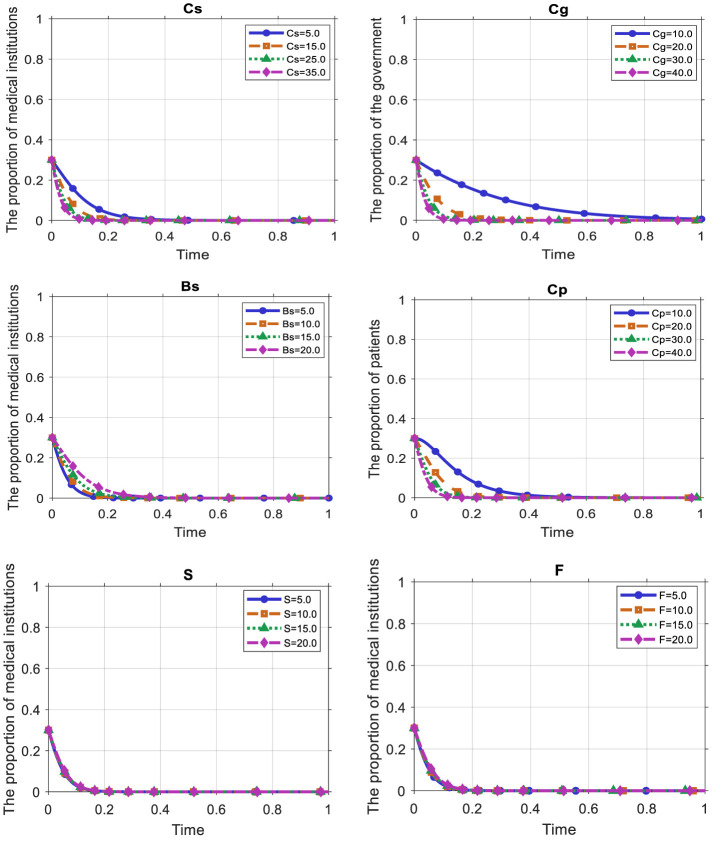
Analysis of key parameters' impact on system stability under pessimistic scenario.

In this scenario, healthcare institutions face high initial costs for data sharing, patients express significant privacy concerns, and government oversight presents considerable challenges, placing the initiative in the early, challenging phase of advancing medical examination data sharing and mutual recognition. The impact of key parameters on the system's steady state is illustrated in Figure 7.

As shown in Figure 7, under the pessimistic scenario, the system exhibits high costs and low returns, with variations in parameters significantly impacting its convergence. An increase in the data sharing cost *C*_*s*_ for medical institutions directly weakens their willingness to share. When *C*_*s*_ rises from 5 to 35, the proportion of institutions sharing data rapidly declines, causing the system to quickly converge toward the suboptimal equilibrium state where “all three parties opt out.” An increase in the government regulatory cost *C*_*g*_ further exacerbates this trend. Even when *C*_*g*_ increases from 10 to 40, the proportion of government regulation approaches zero, indicating that regulatory mechanisms struggle to function effectively in a high-cost environment. Although increasing the direct sharing benefits *B*_*s*_ for medical institutions can marginally improve the sharing rate, its positive impact is offset by high costs in pessimistic scenarios. Even when *B*_*s*_ increases from 5 to 20, the sharing rate fails to rise, unable to alter the system's fundamental convergence toward a negative equilibrium. Increasing patient privacy costs (*C*_*P*_) directly reduces patient participation willingness, when *C*_*P*_ rises from 10 to 40, the patient acceptance rate drops from 0.3 to near zero, creating a vicious cycle. The effects of sharing subsidies (*S*) and non-sharing penalties (*F*) are similar: while increasing their values brings marginal improvements, they cannot reverse the convergence direction when the system's overall benefit is negative.

This parameter sensitivity holds significant practical implications for medical data sharing and mutual recognition. First, under pessimistic scenarios, relying solely on incentives like increased subsidies or penalties struggles to break system lock-in. Instead, reducing cost burdens for all stakeholders at the source is essential. Specifically: Lower *C*_*s*_ by advancing medical data standardization and building regional integrated information platforms. Reduce *C*_*g*_ through intelligent monitoring technologies and streamlined regulatory processes. Minimize *C*_*P*_ by strengthening data security protections and refining privacy safeguards. Second, regarding benefit distribution, a more refined interest-adjustment mechanism is needed to ensure that the shared benefits *B*_*s*_ for medical institutions remain at a reasonable level. Simultaneously, patient satisfaction should be enhanced by improving healthcare service quality and optimizing diagnostic and treatment processes. Finally, policy formulation should emphasize the synergistic effects of various parameters, avoiding excessive adjustments to any single parameter. Through systematic cost control and benefit enhancement strategies, the parameter structure under the pessimistic scenario can be gradually altered, creating conditions for the system to evolve toward a more optimal equilibrium.

(2) Baseline scenario

**Figure 8:**
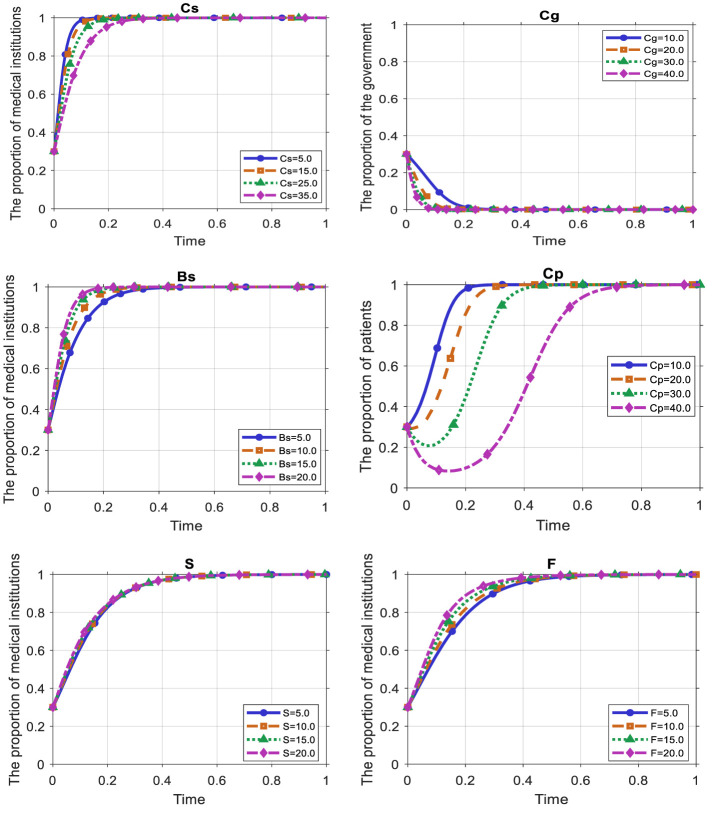
Analysis of key parameters' impact on system stability under baseline scenario.

In this scenario, medical examination data sharing and mutual recognition operates at an intermediate level of cost and benefit, reflecting a development stage where costs and benefits are balanced. The impact of key parameters on the system's stable state is illustrated in Figure 8.

The baseline scenario, as an intermediate state, exhibits heightened sensitivity to parameter variations in system convergence. An increase in the data sharing cost *C*_*s*_ for healthcare institutions significantly slows convergence speed. When *C*_*s*_ rises from 5 to 35, the time required to converge to the active sharing state markedly extends, though the system maintains its evolutionary trajectory toward (0,1,1). Changes in government regulatory cost *C*_*g*_ directly influence government strategy selection. Increasing *C*_*g*_ from 10 to 40 reduces the government's regulatory proportion from 0.3 to near zero, indicating that in this scenario, the government tends to prioritize cost savings by choosing not to regulate. Increasing the direct benefit sharing *B*_*s*_ for medical institutions significantly promotes system convergence. Raising *B*_*s*_ from 5 to 20 markedly increases the medical institution sharing ratio and substantially enhances convergence stability. An increase in patient privacy cost *C*_*P*_ weakens but does not completely break patient participation willingness. When *C*_*P*_ rises from 10 to 40, the patient acceptance ratio shows a downward trend, and the system convergence speed slows accordingly, indicating that patient acceptance still requires a certain amount of time. Sharing subsidies (*S*) and non-sharing penalties (*F*) exhibit similar regulatory effects. Increasing their values effectively accelerates system convergence toward the ideal state. When *S* or *F* increases from 5 to 20, the proportion of sharing by medical institutions rises markedly, and convergence stability is reinforced.

The parameter response characteristics in this scenario offer insights into medical data sharing and mutual recognition: First, prioritize optimizing cost structures by reducing *C*_*s*_ through technological innovation and management improvements. Crucially, narrow the cost gap between active and passive sharing to create favorable conditions for institutions adopting active sharing strategies. Second, improving the benefit distribution mechanism is essential. Research indicates an optimal allocation ratio that ensures both patients and institutions receive reasonable returns. This requires establishing scientific models for benefit calculation and distribution to sustain the willingness of both parties to share. Regarding the government's role, while the baseline scenario achieves an optimal state without government oversight, appropriately reducing regulatory costs (*C*_*g*_) and enhancing government data governance capabilities can accelerate system convergence and strengthen stability. Furthermore, advancing privacy protection technologies is particularly crucial. By reducing the probability of privacy breaches, the negative impacts of increased patient privacy costs (*C*_*P*_) can be effectively mitigated, safeguarding system convergence. The combined implementation of these measures can propel the baseline scenario toward a more stable ideal state, enabling the sustainable development of medical examination data sharing and mutual recognition.

(3) Optimistic scenario

**Figure 9:**
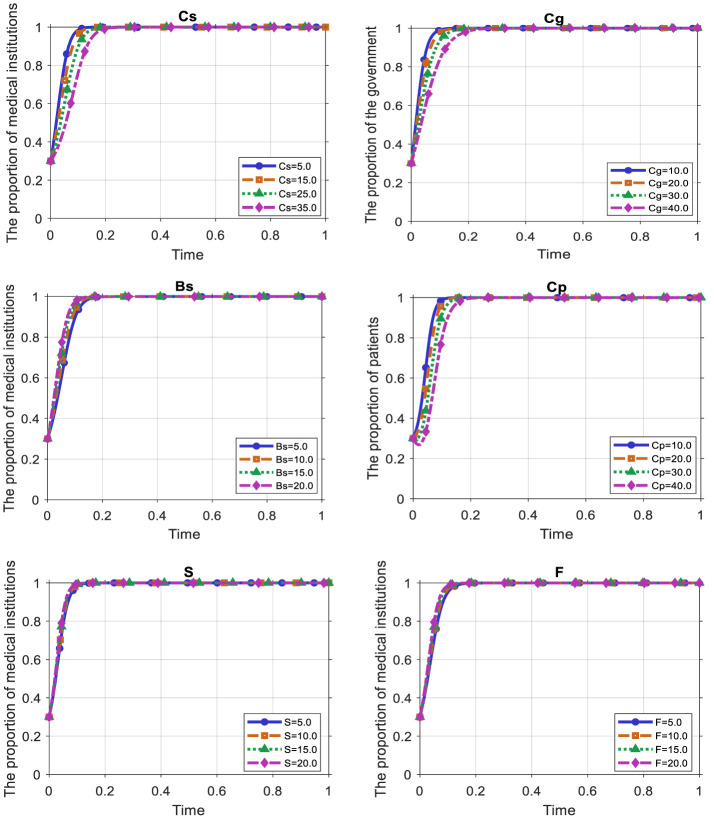
Analysis of key parameters' impact on system stability under optimistic scenario.

In this scenario, medical examination data sharing and mutual recognition, operating under conditions of mature sharing technology and high patient acceptance, reflects a low-cost, high-return mature phase. The impact of key parameters on the system's steady state is illustrated in Figure 9.

In the optimistic scenario, the system exhibits strong robustness. Changes in parameters across a wide range do not alter the ultimate equilibrium of convergence toward (1,1,1), affecting only the convergence speed. An increase in the data sharing cost *C*_*s*_ for medical institutions slows but does not disrupt the convergence process. Even when *C*_*s*_ reaches 35, the proportion of sharing among medical institutions remains close to 1, indicating the system's strong resilience to rising costs. Changes in government regulatory cost *C*_*g*_ exhibit similar characteristics. Although increasing *C*_*g*_ from 10 to 40 slows the growth rate of government oversight, regulatory strategy remains an evolutionarily stable choice, reflecting the system's tolerance for rising regulatory costs. Enhancing the direct sharing benefit *B*_*s*_ for medical institutions accelerates system convergence, but its marginal effect is relatively limited in the already high-benefit optimistic scenario. Increases in patient privacy cost *C*_*P*_ exert the least impact on the system. When *C*_*P*_ varies within the [10,40] range, the patient acceptance ratio consistently remains above 0.8, demonstrating the system's strong adaptability to fluctuations in privacy costs. The primary role of sharing subsidy *S* and non-sharing penalty *F* lies in accelerating convergence; increasing their values further shortens the time required for the system to reach a stable state.

The parameter characteristics of the optimistic scenario provide crucial insights for advanced stages of medical data sharing and mutual recognition. First, realizing this scenario requires favorable initial conditions, including high participation willingness among stakeholders, robust infrastructure, and effective incentive mechanisms. Under these premises, the system's self-reinforcing mechanism ensures stable tripartite collaboration, maintaining optimal performance even amid parameter fluctuations. Second, enhancing the system's resilience against disturbances is essential, achieved by establishing flexible adjustment mechanisms to address potential parameter changes. For instance, while the system demonstrates strong resilience to cost increases, sustained technological advancement and management optimization remain essential to preserve cost advantages. Although fluctuations in privacy costs have limited impact, continuous innovation in privacy-enhancing technologies is crucial to reinforce patient trust.

At the policy level, efforts should focus on building sustainable collaborative mechanisms. This involves leveraging the facilitating role of government oversight, the primary role of medical institutions in sharing data, and the supporting role of patient participation to foster a virtuous cycle of interaction among all three parties. Finally, the realization of the optimistic scenario signifies the maturation of medical examination data sharing and mutual recognition. At this stage, greater emphasis should be placed on refined system management and quality enhancement. Through parameter optimization and mechanism refinement, the system should evolve toward greater efficiency, stability, and sustainability, ultimately maximizing the value of medical data and optimizing public services.

## 6 Discussion and recommendations

### 6.1 Discussion of research findings

This paper employs a tripartite evolutionary game model and numerical simulation to deeply explore the dynamic evolution patterns of medical examination data sharing and mutual recognition systems, providing theoretical foundations and empirical support for addressing the four core questions raised in the introduction.

First, regarding the “conditions under which medical institutions transition from mutual lockdown to sharing and mutual recognition,” simulation results indicate that the key lies in ensuring the net benefit of sharing is positive and significantly exceeds the benefit of lockdown. The specific threshold condition is: (*B*_*s*_ + *B*_*n*_ − *C*_*s*_) + *x*(*S* + *F*) > *L*_*d*_. This finding aligns closely with Hangzhou's dual-pronged strategy of medical insurance incentives (10.0978 million yuan) and technological cost reduction (18). The study also reveals that network effect benefits *B*_*n*_ exhibit exponential growth over time, starting slowly before accelerating once a critical mass is reached. This explains the “start-up difficulty” observed in mutual recognition adoption.

Second, regarding the “threshold for effective government regulation,” this study finds that the critical condition for proactive government oversight is *y*(*T* − *C*_*g*_ − *S* − *L*_*g*_) > (*C*_*g*_ − *F*_*g*_). That is, the interaction effect between policy reputation gains and shared behavior among medical institutions must exceed net regulatory costs. Under pessimistic scenarios, this condition fails to hold, leaving the government lacking regulatory motivation. Conversely, under optimistic scenarios, the condition is fully satisfied, driving strong regulatory intent from the government.

Jining City, Shandong Province, implemented digital oversight measures by establishing an integrated city-wide “mutual recognition cloud platform.” This platform enables automatic data aggregation, retrieval, and intelligent early warning systems, replacing traditional manual inspections. Simultaneously, it established long-term mechanisms including “red-yellow-blue” inspections, data reporting, and third-party evaluations, incorporating mutual recognition quality into medical institutions' performance assessments, significantly reducing government oversight costs (35). Hainan Province, meanwhile, adopted advanced technical approaches for medical insurance data governance. Based on “trusted data space” technology, it achieved secure collaboration where “data remains within domains, usable but not visible,” mechanically reducing regulatory complexity and risks (36). These cases demonstrate that reducing regulatory costs *C*_*g*_ through digital oversight tools and enhancing policy credibility *T*—by integrating medical test mutual recognition into performance evaluations—is key to improving regulatory effectiveness. Research also reveals strategic substitution effects in government oversight: when medical institutions exhibit high self-discipline, government oversight can gradually withdraw; when patient engagement is high, regulatory intensity can be appropriately reduced.

Third, regarding the design of “medical insurance payment incentives,” simulation results show dynamic incentive mechanisms outperform fixed subsidies. For instance, linking subsidies *S* to mutual recognition quality (*S*= S_0_ × mutual recognition index) or penalties *F* to violation severity (*F* = *F*_0_× number of violations) significantly enhances policy efficiency. Hangzhou's practice validates this approach, as its “performance evaluation and targeted incentive” system dynamically rewards based on the quantity and quality of mutual recognition. The study also found that the timing of incentives is crucial: during the early stages of system evolution, subsidies should be the primary tool supplemented by penalties; as the system approaches stability, the proportion of subsidies should be gradually reduced, shifting toward penalties to maintain institutional rigidity.

Fourth, regarding “institutional innovation for data as a factor of production,” this study highlights the importance of defining data property rights and establishing revenue distribution mechanisms. A clear data property rights system can reduce privacy costs *C*_*P*_—such as through anonymization techniques; reasonable benefit distribution can enhance network effect benefits *B*_*n*_—such as through data value mining. This study further proposes that the data trust model may serve as a governance innovation for medical data sharing. By having a third-party professional institution manage data usage rights and benefit distribution, it can both safeguard patient privacy rights and promote the circulation of data value.

### 6.2 Policy recommendations

Based on the above research findings, this paper proposes the following policy recommendations:

First, implement a tiered advancement strategy. Adopt differentiated approaches tailored to distinct developmental stages and regional characteristics. In pessimistic scenario regions, such as economically underdeveloped areas, prioritize government-led initiatives to activate the system through strong incentives (high *S*) and stringent oversight (high *F*). In baseline scenario regions (e.g., moderately developed areas), pursue a government-market collaboration strategy focused on reducing sharing costs *C*_*s*_. Optimistic scenario regions (e.g., developed areas) may transition to market-driven models, shifting the government's role from pre-approval to post-event oversight. It is recommended that the state select eligible cities and prefectures for pilot programs to develop replicable and scalable models.

Second, establish a multi-faceted incentive mechanism. Drawing from Hangzhou's integrated incentive system—combining medical insurance incentives, performance evaluations, and special rewards—economic incentives should be paired with non-economic incentives (e.g., reputation assessments, priority in accreditation reviews). Establish a dynamic adjustment mechanism to align incentive intensity with mutual recognition effectiveness. Actively explore innovative systems like “mutual recognition liability insurance” to alleviate concerns for medical institutions. For patient participation, consider implementing a “health points” system linking data sharing to personalized health management services, enhancing the sense of gain from participation.

Third, strengthen technological empowerment and standardization to advance the interoperability and user-friendliness of mutual recognition platforms, lowering technical barriers. Develop unified data standards, quality standards, and security standards to provide a technical foundation for mutual recognition. Strengthen the information technology capabilities of medical institutions to improve the standardization of data collection and utilization. It is recommended that the National Health Commission take the lead in establishing nationally unified technical standards and interface specifications for medical examination data sharing to prevent the emergence of new “data silos.”

Fourth, improve legal frameworks and governance systems by clarifying data ownership, usage rights, and revenue rights, while strengthening privacy protection and liability determination mechanisms. Establish a multi-departmental collaborative governance structure, such as a coordinated working mechanism involving medical insurance, finance, and data authorities, to formulate clear mutual recognition rules and exception scenarios, thereby safeguarding medical quality and safety. It is recommended to revise the Regulations on the Administration of Medical Institutions to mandate mutual recognition of examination and testing results as a legal obligation for medical institutions, accompanied by corresponding liability exemption clauses.

### 6.3 Research limitations and outlook

This study has several limitations. On one hand, it assumes agents are perfectly rational, whereas real-world decision-making may involve bounded rationality and emotional factors. On the other hand, it primarily considers macro-level factors without delving into micro-level motivational mechanisms within organizations. Future research could incorporate bounded rationality assumptions and explore micro-level incentive and constraint mechanisms within organizations to address these shortcomings.

Future research may incorporate prospect theory or mental accounting theory to more accurately describe decision-making behaviors. Empirical testing and parameter calibration using real-world data could enhance model predictive accuracy. Concurrently, exploring application models and governance mechanisms for emerging technologies like blockchain in medical examination mutual recognition is warranted. As the process of medical data commoditization accelerates, research on medical examination data sharing and mutual recognition will provide sustained theoretical support and practical guidance for building an efficient and secure medical data circulation environment.

## Data Availability

The original contributions presented in the study are included in the article/supplementary material, further inquiries can be directed to the corresponding author.
